# Increased monocyte abundance as a marker for relapse after discontinuation of biologics in inflammatory bowel disease with deep remission

**DOI:** 10.3389/fimmu.2022.996875

**Published:** 2022-11-01

**Authors:** Yiyoung Kwon, Yoon Zi Kim, Yon Ho Choe, Mi Jin Kim

**Affiliations:** Department of Pediatrics, Samsung Medical Center, Sungkyunkwan University School of Medicine, Seoul, Korea

**Keywords:** Crohn’s disease, ulcerative colitis, children, monocytes, biologics, relapse, remission

## Abstract

Monocytes are involved in the upstream inflammatory process in the immune reaction in inflammatory bowel disease (IBD). Patients with IBD who discontinued biologics have been found to relapse, even after checking for deep remission. This study investigated whether monocytes could act as a predictor of relapse in patients who experienced relapse after the discontinuation of biologics. To this end, pediatric patients (<19 years old, n = 727) diagnosed with IBD from January 2003 to December 2021 were retrospectively reviewed. Clinical features, monocytes, and disease activity at the time of discontinuing biologics were evaluated by dividing patients into a relapsed group and a non-relapsed group after discontinuing biologics. The percentage of monocytes (8.65% vs. 6.42%, P < 0.001), the absolute monocyte count (614.79 cells/μL vs. 381.70 cells/μL, P < 0.001), and the monocyte/polymorphonuclear leukocyte (PMN) ratio (0.18 vs. 0.11, P < 0.001) at the time of discontinuation were significantly higher in patients who experienced relapse. As a result of multivariate analysis, the monocyte percentage (odds ratio: 2.012, P < 0.001) and monocyte/PMN ratio (odds ratio: 4.320E+14, P = 0.002) were evaluated as risk factors for relapse. Diagnostic capability was confirmed using area under operating characteristic curve (0.782) of the monocyte percentage for assessing the relapse within 6 months with cutoff value of 8.15% (P < 0.001). The findings presented in this study indicate that the patients with high monocyte counts experienced relapse after the discontinuation of biologics. A monocyte percentage of over 8.15% in the blood at the time of discontinuation was found to be associated with a high probability of relapse within 6 months, even in deep remission.

## Introduction

**Figure 1:**
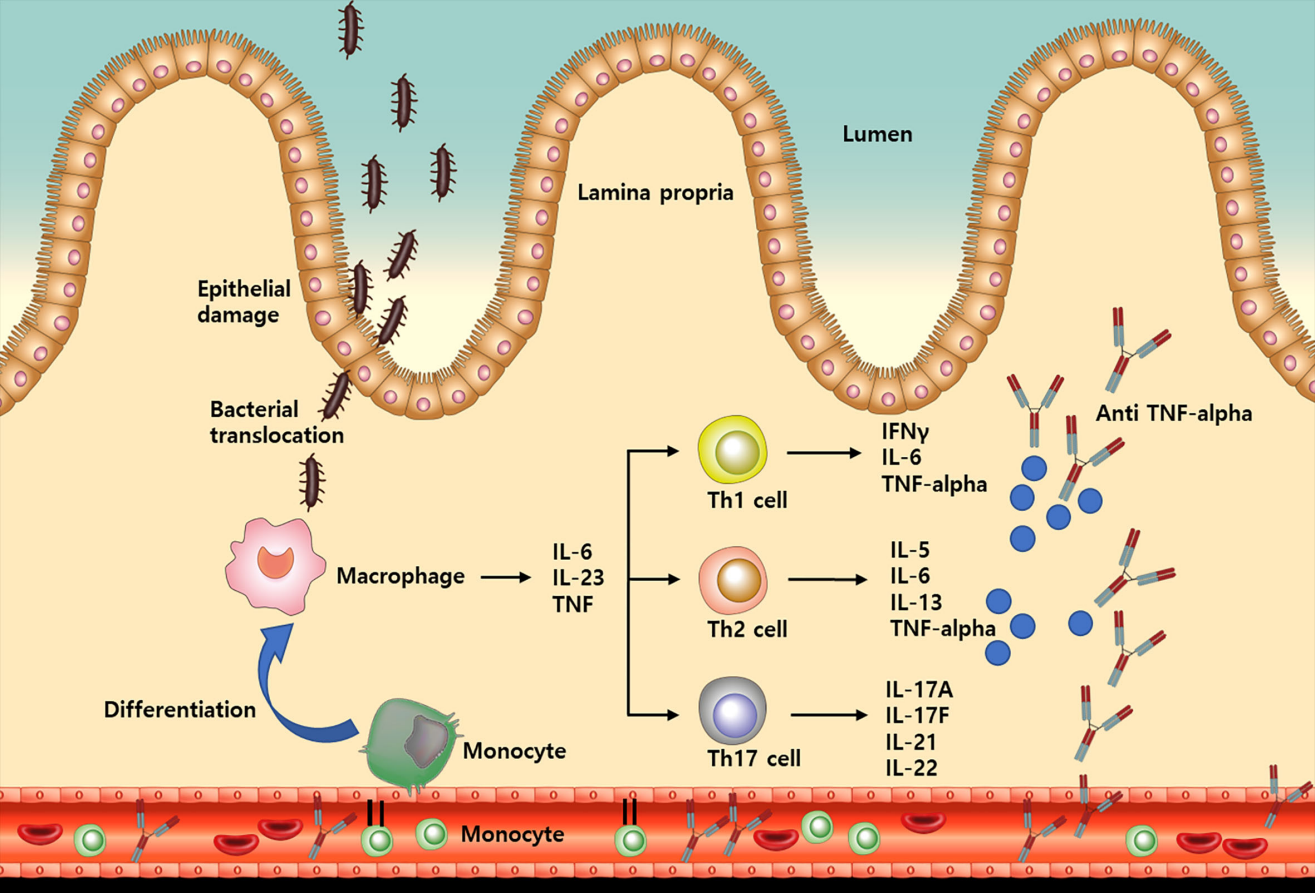
A simplified illustration of the process from monocyte recruitment to TNF-alpha release in inflammatory bowel disease. IL, Interleukin; Th, T helper; TNF, Tumor necrosis factor; IFN, Interferon.

Inflammatory bowel disease (IBD) is a lifelong chronic disease, the potential causes of which include immune system dysfunction (1). Innate immune abnormalities lead to adaptive immune disorders in the gut, which have been characterized by T helper and regulatory cell imbalance (2, 3). Monocytes are a subset of circulating white blood cells and involved in the innate immune system. They are recruited from the bone marrow to gut tissue and differentiate into macrophage and dendritic cells, which produce inflammatory cytokines and chemokines for downstream process of inflammation in IBD (**Figure 1**) (4–6).

Since monocytes are located in the most upstream of the inflammatory process, studies have been conducted to analyze the association between monocytes and IBD. Koch et al. (7) investigated the role of proinflammatory CD16^+^ monocytes in the pathogenesis of IBD and found that the abundance CD16^+^ monocytes increased significantly in Crohn’s disease (CD). In a recent study, Anderson et al. (8) evaluated monocytosis as a biomarker of severity in IBD and found that IBD patients with monocytosis were at increased risk of worse clinical outcomes. Another recent study by Furukawa et al. (9) demonstrated the association between the blood monocyte count and mucosal healing in patients with ulcerative colitis (UC). Their results indicated that monocyte count may be used as a marker for mucosal healing in patients with low C-reactive protein (CRP) levels.

Since the introduction of biologics, the systemic use of steroids in IBD patients has reduced, and an increase in the rate of clinical remission has been observed. However, the precise endpoint of biologics administration and whether remission can be maintained without treatment remain a matter of debate. Even after deep remission, clinical cases of relapse in patients who discontinued biologics have been reported, with a large number of the relapsed patients having to restart treatment (10, 11). In fact, one study suggested that combined clinical and endoscopic remission should not be an impetus to consider therapeutic de-escalation (12).

Although treatment is often discontinued after confirming deep remission, there are several reports of relapse in patients administered biologics, with some patients relapsing rapidly within 6 to 12 months (10). In addition, monocytosis has been observed in patients with normal erythrocyte sedimentation rate (ESR) and CRP after achieving remission during treatment with biologics. Thus, as monocytes are known to play a role in upstream inflammation processes, monocytosis can be observed even in patients with normal ESR and CRP levels by suppressing the lower stage of inflammation by biologics. The purpose of this study was to evaluate the monocytes of patients who relapsed after the discontinuation of biologics compared to patients who did not.

## Methods

### Patients and study design

**Figure 2:**
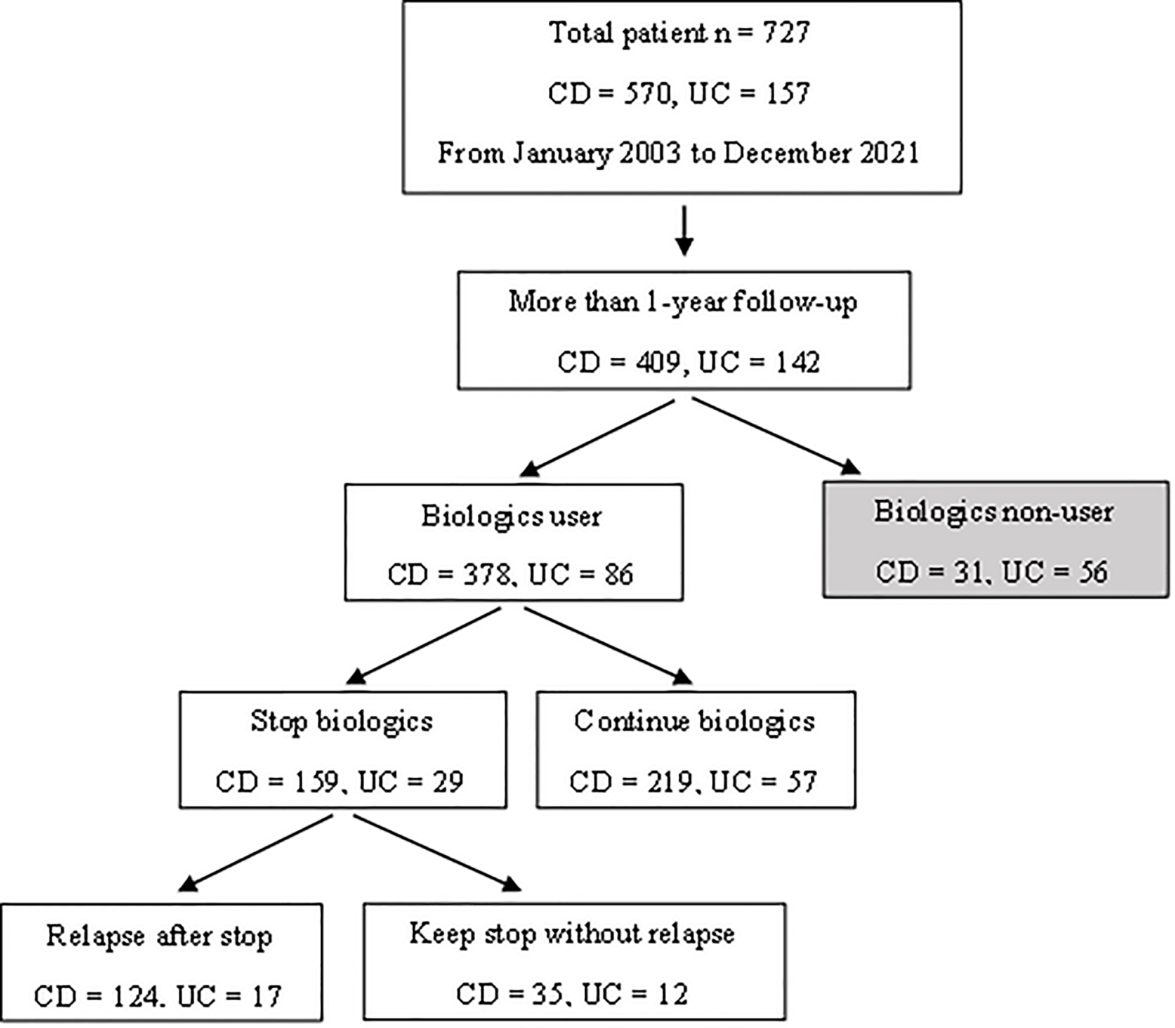
Flow chart illustrating the subject selection process and patient characteristics.

This study retrospectively reviewed 727 pediatric patients (<19 years old) diagnosed with IBD from January 2003 to December 2021. CD and UC was diagnosed following the guidelines of the European Society for Pediatric Gastroenterology, Hepatology and Nutrition (the Porto criteria) (13). Among the patients enrolled, 378 CD and 86 UC patients had been administered biologics with a follow-up period of more than 12 months. A further 159 CD and 29 UC patients had discontinued biologics after confirming deep remission. Oral medications that were being taken at the time the biologics were discontinued were not discontinued. All patients were subjected to laboratory testing and endoscopic procedures, and 124 CD and 17 UC patients experienced clinical relapse after the discontinuation of biologics (**Figure 2**).

Follow-ups were performed at an outpatient clinic every 4 to 8 weeks according to the biologics insurance standard with laboratory tests (complete blood count with differential, chemistry profile, ESR, CRP, amylase, and lipase) performed at each visit. The patients were recommended to submit stool samples every 3 to 4 months for calprotectin testing. Most of the patients underwent both esophagogastroduodenoscopy and colonoscopy at the time of diagnosis and after 12 months of biologics treatment. Thereafter, endoscopy was performed every 2 years. Once patients had normal laboratory tests and calprotectin results, the discontinuation of biologics was planned and endoscopy was re-executed to confirm ER status.

The purpose of this study was to compare and evaluate the monocytes of pediatric IBD patients who experienced clinical relapse (124 CD and 17 UC patients) after the discontinuation of biologics against patients who did not (35 CD and 12 UC patients). If monocytes were found to act as a risk factor for relapse, the second objective of this study was to identify the optimal cutoff value of monocytes as an indicator of whether to discontinue biologics. All methods were performed in accordance with the relevant guidelines and regulations and were approved by the Clinical Research Ethics Committee of Samsung Medical Center (IRB file no. SMC 2022-04-062).

### Data collection

Data collected at diagnosis included age, sex, laboratory results (hematocrit, albumin, ESR, and CRP), pediatric Crohn’s disease activity index (PCDAI), pediatric ulcerative colitis activity index (PUCAI), and extent and severity of disease. Based on the colonoscopy findings, the extent of disease and characteristics of CD and UC were classified according to the Paris classification, while severity was classified using the simple endoscopic score for Crohn’s disease (SES-CD) score for CD patients and the Mayo endoscopic subscore (MES) for UC patients. Information on the use of medication for IBD, including systemic steroids, immunosuppressants (azathioprine, methotrexate, and cyclosporine), and biologics (infliximab and adalimumab), were also recorded.

Data collected at the time of biologic discontinuation included laboratory results, colonoscopy results, PCDAI, and PUCAI again, as well as data related to calprotectin, medication status, and monocytes. Complete blood counts were all measured with the same equipment (Sysmex XN, Sysmex Europe, Hamburg). When collecting data for monocytes, the percentage of monocytes in the white blood cell (WBC) count (normal range: 2.0–8.0%), the absolute monocyte count (normal range: 200–800/μL), and the ratio of monocytes divided by polymorphonuclear leukocytes (PMN) were recorded. Data of the laboratory results and monocyte count at 2 months before relapse were also collected. Since ESR and CRP are typically elevated beyond the normal range at the time of relapse, data were collected two months before relapse to evaluate whether monocytes revealed abnormal results, indicative of the possibility of relapse, at a time when the ESR and CRP values were normal. We also collected data of other blood tests (ESR and CRP) and monocytes, the data collection for which was performed on the same day.

### Assessment of remission and relapse

Deep remission was defined as achieving both clinical remission (CR) and endoscopic remission (ER). For CD patients, CR was defined as PCDAI ≤ 10 and ER was defined as a SES-CD score of 0–2 (14). Although there is no formal definition of deep remission in relation to UC, the same standard as that for the CD patients was applied. CR was defined as PUCAI < 10, and ER was defined as MES of 0–1.

Relapse was defined as clinical relapse. Clinical relapse was defined as PCDAI ≥ 10 for CD patients and PUCAI >10 for UC patients with modification of treatment. Cases in which symptoms temporarily worsened due to infections, such as gastroenteritis or colitis, and CMV infection were excluded.

### Statistical analysis

**Figure 3:**
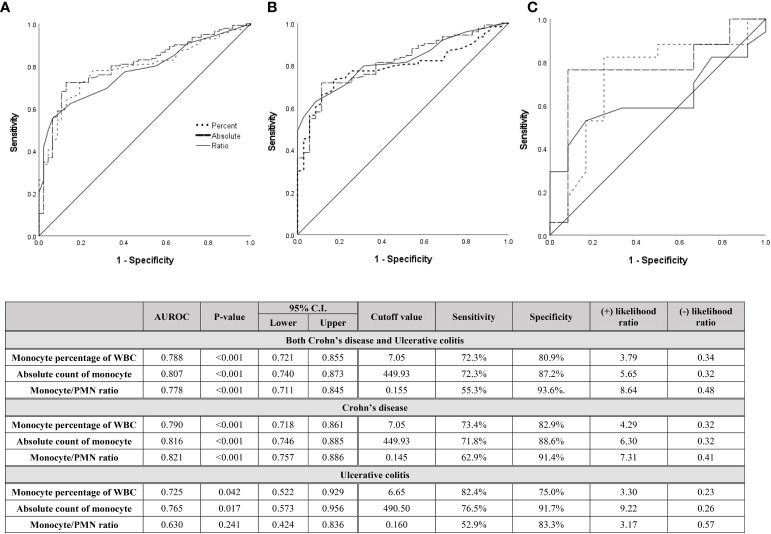
Diagnostic capabilities with area under operating characteristic curve of the serum monocyte for assessing relapse in **(A)** both CD and UC patients, **(B)** only CD patients, and **(C)** only UC patients after discontinuation of biologics.

**Figure 4:**
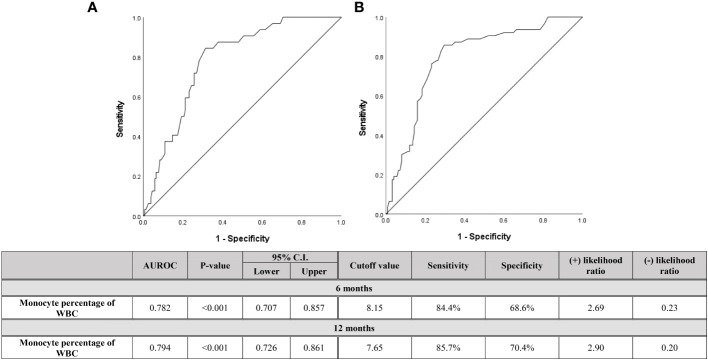
Diagnostic capabilities with area under operating characteristic curve of the serum monocyte percentage of WBC for assessing relapse within **(A)** 6 months and **(B)** 12 months after discontinuation of biologics in both pediatric CD and UC patients.

For descriptive statistics, continuous variables were expressed as the mean ± standard deviation (SD), while categorical variables were expressed as a percentage. Student’s t-test and the Mann-Whitney U test were used to compare groups. Pearson correlation analysis was used to analyze the correlation between monocytes and other variables (**Table 3**). Univariate and multivariate logistic regression analysis were used to evaluate risk factors for relapse (**Table 4**). The diagnostic capability of monocytes was evaluated using the receiver operating characteristic (ROC) curve (**Figures 3** and **4**). Diagnostic performance was expressed as the diagnostic sensitivity, specificity, and area under the ROC curve. Youden’s J statistic was used to calculate cutoff values. Kaplan-Meier survival plots were used for descriptive time-dependent data (**Figure 6**). All the above statistical analyses were performed using SPSS version 27 (IBM Corporation, Armonk, NY, USA). P < 0.05 was considered statistically significant.

## Results

### Comparing clinical features at diagnosis between relapsed and non-relapsed patients

The basal clinical characteristics at the time of diagnosis of the two groups, i.e., patients who experienced relapse (124 CD and 17 UC patients) after the discontinuation of biologics and those who did not (35 CD and 12 UC patients), were compared (**Table 1**). When CD patients were evaluated, demographic features such as the mean age, sex proportion, and total duration of follow-up period were not significantly different (P = 0.656, 0.320, and 0.062, respectively). The PCDAI score, laboratory results at diagnosis, and initial usage of medication (5-aminosalicylic acid (5-ASA), systemic steroids, and immunomodulators) in the two groups were also not significantly different. When Paris classification was evaluated, the diagnosis age of the majority of both groups was A1b (10 to <17 years). Only location evaluation revealed a significant difference between the two groups; the majority of relapsed patients (79.8%) involved both the ileum and colon (L3), whereas patients without relapse had a relatively high proportion of patients with only the ileum (L1) or colon (L2) being involved (P = 0.020). These results show that patients with more tissues affected are more prone to relapse. No significant difference was observed between the two groups in terms of behavior or growth. The evaluation of disease severity with SES-CD score was also similar (P = 0.991).

**Table 1:** Demographic and clinical features at diagnosis of the two experimental groups: patients who experienced relapse and patients who did not experience relapse after biologic discontinuation.

	Crohn’s disease (N = 159)		Ulcerative colitis (N = 29)		P-value	
	Relapse (N = 124)	No relapse (N = 35)	Relapse (N = 17)	No relapse (N = 12)	CD	UC
Age at diagnosis, years	14.23 ± 2.74	14.51 ± 3.39	14.75 ± 2.53	14.13 ± 2.48	0.656^e^	0.521^e^
Male/ female	96 (77.4%) / 28 (22.6%)	24 (68.6%)/ 11 (31.4%)	7 (41.2%)/ 10 (58.8%)	8 (66.7%)/ 4 (33.3%)	0.320^f^	0.188^f^
Total duration of follow-up, years	13.02 ± 4.32	10.42 ± 2.95	7.31 ± 2.54	11.00 ± 3.41	0.062^e^	**0.005** ^e^
PCDAI^a^	33.14 ± 14.27	38.00 ± 11.51	–	**-**	0.424^e^	**-**
PUCAI^b^	–	–	56.76 ± 17.22	37.92 ± 11.96	^-^	**0.002** ^e^
Hematocrit, g/dl	36.17 ± 6.29	35.24 ± 4.48	32.03 ± 8.02	36.02 ± 5.05	0.331^e^	0.112^e^
Albumin, g/dl	3.79 ± 0.56	3.75 ± 0.56	3.96 ± 0.66	4.39 ± 0.33	0.725^e^	**0.045** ^e^
ESR, mm/h	15.17 ± 17.22	9.63 ± 9.23	35.71 ± 28.72	16.33 ± 17.50	0.078^e^	**0.033** ^e^
CRP, mg/dl	2.80 ± 2.89	3.34 ± 3.12	1.02 ± 1.84	0.14 ± 0.19	0.363^e^	0.112^e^
Medication						
5-ASA	115 (92.7%)	34 (97.1%)	17 (100%)	12 (100%)	0.237^f^	–
Systemic steroids	21 (16.9%)	10 (28.6%)	13 (76.5%)	4 (33.3%)	0.127^f^	**0.024** ^f^
Immunomodulators	122 (98.4%)	34 (97.1%)	17 (100%)	12 (100%)	0.688^f^	–
CD age						
A1a (0-<10 years old)	4 (3.2%)	2 (5.7%)		**-**	0.677^f^	**-**
A1b (10-<17 years old)	103 (72.5%)	26 (74.3%)				
A2 (17–40 years old)	17 (13.7%)	7 (20%)				
CD location						
L1 (Ileal)	16 (11.3%)	8 (22.9%)		**-**	**0.020** ^f^	**-**
L2 (Colonic)	9 (7.3%)	7 (20.0%)				
L3 (Ileocolonic)	99 (79.8%)	20 (57.1%)				
L4a (Proximal upper involvement)	49 (39.5%)	14 (40.0%)				
L4b (Distal upper involvement)	64 (51.6%)	14 (40.0%)				
CD behavior						
B1 (Inflammatory)	93 (75.0%)	24 (68.6%)		–	0.654^f^	–
B2 (Stricturing)	24 (19.4%)	11 (3.4%)				
B3 (Penetrating)	5 (4.0%)	0				
P (Perianal disease)	85 (68.5%)	24 (68.6%)				
CD Growth						
G0 (No evidence of delay in growth)	35 (28.2%)	9 (25.7%)			0.769^f^	**-**
G1 (Delay in growth)	89 (71.8%)	26 (74.3%)				
SES-CD^c^ score	16.95 ± 8.89	16.97 ± 9.24			0.991^e^	**-**
UC extent						
E1 (Proctitis)	–		0	4 (33.3%)	**-**	**0.036** ^f^
E2 (Left colitis)			1 (5.9%)	0		
E3 (Extensive colitis)			2 (11.8%)	1 (8.3%)		
E4 (Pancolitis)			14 (82.4%)	7 (58.3%)		
UC MES^d^						
1 (Mild)	–		0	1 (8.3%)	–	**0.007** ^f^
2 (Moderate)			8 (47.1%)	10 (83.3%)		
3 (Severe)			9 (52.9%)	1 (8.3%)		

Values are n (percentage) or average ± standard deviation; N, number of patients.

a Pediatric Crohn’s disease activity index (PCDAI) score can range from 0 to 100, with higher scores signifying more active disease. A score of <10 indicates consistent with inactive disease, 11–30 indicates mild disease, and >30 indicates moderate-to-severe disease.

b Pediatric Ulcerative Colitis Activity Index (PUCAI) is a 6-item disease activity index intended for use in pediatric UC clinical trials with a score ranging from 0 to 85.

c The simple endoscopic score for Crohn’s disease (SES-CD) assesses the size of mucosal ulcers, the ulcerated surface, the endoscopic extension, and the presence of stenosis.

d Mayo endoscopy subscores were as follows: 0, normal or inactive disease; 1, mild disease; 2, moderate disease; and 3, severe disease.

e Student’s t-test.

f Mann-Whitney U test.

Bold values mean statistically significant values with p-value <0.05.

When UC patients were evaluated, demographic features such as the mean age and sex proportion were not significantly different (P = 0.521 and 0.188, respectively). Although the total duration of the follow-up period was significantly different, the follow-up period of patients without relapse was longer (7.31 years vs. 11.00 years with P = 0.005). Unlike CD patients, relapse in UC patients was associated with a significantly higher PUCAI score at the time of diagnosis compared to patients who did not relapse (56.76 vs. 37.92 with P = 0.002), which was supported by the laboratory results. In the relapsed patient group, albumin was significantly lower (3.96 vs. 4.39 with P = 0.045) and ESR was higher (35.71 vs. 16.33 with P = 0.033) at the time of diagnosis. In addition, although no difference was observed in other medications, the frequency of systemic steroid use was high in the relapsed patient group (76.5% vs. 33.3% with P = 0.024). As evidenced by the endoscopic evaluation, the rate of pancolitis was uniquely high (82.4% vs. 58.3%) in the relapsed patient group, and conversely, the rate of proctitis was high in the patient group without relapse. As with CD, patients with more tissues affected tend to have more relapse. Even in the MES evaluation, the proportion of patients with severe condition was significantly higher in the relapsed patient group (52.9% vs. 8.3% with P = 0.007).

### Evaluation of monocytes at the time of biologic discontinuation and subsequent relapse

**Table 2** shows the clinical features and laboratory results at the time of biologic discontinuation. All patients discontinued biologics treatment after confirming the ER. In the case of CD, since the definition of ER is the SES-CD score of 0-2, the number of patients corresponding to 0,1,or 2 point was evaluated. The proportion of patients with a score of 0 was higher in patients who don’t experience relapse (58.9% vs. 88.6%) and the proportion of patients with score of 2 was higher in patients with relapse (41.1% vs. 11.4%) (P = 0.001). In the case of UC, ER was evaluated by MES score of 0 or 1 point, and although it was not statistically significant, the proportion corresponding to 1 point was high in patients who experienced relapse like CD. The status of medication at the time of discontinuation was as follows: relapsed individuals were taking more 5-ASA (68.5% vs. 45.7% with P = 0.013) and immunomodulators (mostly azathioprine) (79.8% vs. 62.9% with P = 0.038) in the case of CD patients; in UC patients, immunomodulators (mostly azathioprine) (70.6% vs. 33.3% with P = 0.048) were more frequently used in relapsed patients. All patients were using biologics: UC patients were taking infliximab because infliximab is the only insurance drug for pediatric patients, while CD patients were taking infliximab or adalimumab; however, the proportion of infliximab use was higher than that of adalimumab in both groups.

**Table 2:** Clinical features and monocyte count at discontinuation of biologic agents of the two experimental groups: patients who experienced relapse and patients who did not experience relapse after biologic discontinuation.

	Crohn’s disease (N = 159)		Ulcerative colitis (N = 29)		P-value	
	Relapse (N = 124)	No relapse (N = 35)	Relapse (N = 17)	No relapse (N = 12)	CD	UC
SES-CD^a^ score						
0	73 (58.9%)	31 (88.6%)			
1	0	0				**0.001** ^f^
2	51 (41.1%)	4 (11.4%)				
UC MES^b^						
0			11 (64.7%)	10 (83.3%)		0.266^f^
1			6 (35.3%)	2 (16.7%)		
Medication						
5-ASA	85 (68.5%)	16 (45.7%)	11 (64.7%)	8 (66.7%)	**0.013** ^f^	0.917^f^
Systemic steroids	1 (0.8%)	0	1 (5.9%)	0	0.319^f^	0.332^f^
Immunomodulators	99 (79.8%)	22 (62.9%)	12 (70.6%)	4 (33.3%)	**0.038** ^f^	**0.048** ^f^
Biologics						
Type of biologics at d/c						
- Infliximab	112 (90.3%)	26 (74.3%)	17 (100%)	12 (100%)	0.997^f^	–
- Adalimumab	12 (9.7%)	9 (25.7%)	0	0		
Treatment duration, years	1.93 ± 1.37	2.52 ± 1.72	2.51 ± 2.12	3.07 ± 1.72	**0.037** ^e^	0.440^e^
WBC count, blood, 1 × 10^3^/µL	6.64 ± 1.97	6.80 ± 2.03	7.30 ± 2.07	7.14 ± 1.89	0.680^e^	0.831^e^
PMN count, blood, 1 × 10^3^/µL	4.09 ± 1.58	3.62 ± 1.12	4.42 ± 3.57	3.39 ± 1.38	0.104^e^	0.288^e^
Monocyte	8.65 ± 2.60	6.42 ± 1.13	7.29 ± 1.31	6.18 ± 1.47	**<0.001** ^e^	**0.048** ^e^
Percentage of WBC	614.79 ± 272.07	381.70 ± 103.79	584.34 ± 236.81	418.49 ± 170.41	**<0.001** ^e^	**0.037** ^e^
Absolute count	0.18 ± 0.08	0.11 ± 0.03	0.18 ± 0.11	0.13 ± 0.04	**<0.001** ^e^	0.188^e^
Monocyte/PMN ratio						
PCDAI^c^	5.20 ± 2.06	3.71 ± 2.53	–	**-**	**<0.001** ^e^	**-**
PUCAI^d^	–	–	7.35 ± 9.86	2.50 ± 4.52	–	0.088^e^
Hematocrit, g/dl	41.56 ± 4.33	42.64 ± 4.12	41.56 ± 3.40	41.52 ± 3.29	0.182^e^	0.964^e^
Albumin, g/dl	4.51 ± 0.37	4.61 ± 0.26	4.56 ± 0.26	4.48 ± 0.23	0.140^e^	0.381^e^
ESR, mm/h	15.17 ± 6.22	9.63 ± 9.23	13.59 ± 5.29	8.42 ± 6.16	**0.013** ^e^	0.221^e^
CRP, mg/dl	0.26 ± 0.14	0.10 ± 0.12	0.34 ± 0.12	0.05 ± 0.06	0.070^e^	0.245^e^
Calprotectin, mg/kg	132.16 ± 45.13	90.37 ± 26.74	153.07 ± 41.60	142.34 ± 55.35	**0.011** ^e^	0.643^e^

Values are n (percentage) or average ± standard deviation; N, number of patients.

a The simple endoscopic score for Crohn’s disease (SES-CD) assesses the size of mucosal ulcers, the ulcerated surface, the endoscopic extension, and the presence of stenosis.

b Mayo endoscopy subscores were as follows: 0, normal or inactive disease; 1, mild disease; 2, moderate disease; and 3, severe disease.

c Pediatric Crohn’s disease activity index (PCDAI) score can range from 0 to 100, with higher scores signifying more active disease. A score of <10 indicates consistent with inactive disease, 11–30 indicates mild disease, and >30 indicates moderate-to-severe disease.

d Pediatric Ulcerative Colitis Activity Index (PUCAI) is a 6-item disease activity index intended for use in pediatric UC clinical trials with a score ranging from 0 to 85.

e Student’s t-test.

f Mann-Whitney U test.

Bold values mean statistically significant values with p-value <0.05.

Although PCDAI and PUCAI were within the range corresponding to CR, in the case of CD patients, the PCDAI of relapsed patients showed a significantly higher value (5.20 vs. 3.71 with P < 0.001). ESR was within the normal range (The normal range is 0 to 22 mm/hr for men and 0 to 27 mm/hr for women), and CRP (The normal range is less than 0.5mg/dl) and fecal calprotectin (The normal range is less than 50.0mg/kg) were also within the range, corresponding to biochemical remission at the time of biologic discontinuation in all patients. However, ESR showed significantly higher values even within the normal range in relapsed patients in the case of CD patients (P = 0.013). Fecal calprotectin was elevated outside the normal range (P = 0.011).

With regards to the evaluation of monocytes, the percentage of monocytes in the WBC count (8.65 vs. 6.42 with P < 0.001), the absolute monocyte count (614.79 vs. 381.70 with P < 0.001), and the monocyte/PMN ratio (0.18 vs. 0.11 with P < 0.001) at the time of discontinuation in CD patients were significantly higher in patients who experienced relapse. Even in UC patients, the percentage of monocytes in the WBC count (7.29 vs. 6.18 with P = 0.048) and the absolute monocyte count (584.34 vs. 418.49 with P = 0.037) were significantly higher in those patients who experienced relapse, while the monocyte/PMN ratio was not significant (P = 0.188); however, the relapse group showed a higher mean value (0.18 vs. 0.13).

**Supplementary Figure 2** shows the changes in monocytes at the time of biologic discontinuation and at the time of relapse in CD and UC patients who experienced relapse after the discontinuation of biologics. The monocyte percentage of the WBC count increased between the time of discontinuation and relapse in both CD and UC patients. In the CD patients, the mean value rose from 8.65 to 9.67, which could be considered as a significant increase (P = 0.001). Similarly, in UC patients, the mean value increased from 7.29 to 10.13, which could be considered a significant increase (P < 0.001).

### Correlation analysis between monocytes and variables related to IBD

If monocytes are a significant indicator of the loading of inflammation, we then evaluated whether this was correlated with other factors showing activity in CD and UC (**Table 3**). In addition, we evaluated whether the monocyte values were related to the length of time biologics were administered, as well as whether relapse occurred faster in patients with higher monocyte counts. The monocyte percentage was found to be significantly correlated with albumin, ESR, CRP, and calprotectin (Pearson coefficient of -0.326, 0.299, 0.314, and 0.371 and P < 0.001, < 0.001, < 0.001, and 0.005, respectively), as well as a correlation with the SES-CD score (Pearson coefficient of 0.318 and P < 0.001). An interesting result was the fact that monocytes showed a significant negative correlation with the time taken from biologic discontinuation to the occurrence of relapse (Pearson coefficient = -0.283 and P < 0.001). The absolute monocyte count and monocyte/PMN ratio values also showed significant correlations with albumin, ESR, CRP, calprotectin, and SES-CD scores and were found to be correlated with the PCDAI score. No significant correlation was observed with the duration of biologic administration. Only some of these values were found to be correlated in UC patients. The absolute monocyte count was correlated with ESR, CRP, PUCAI score, and MES (Pearson coefficient of 0.629, 0.721, 0.584, and 0.392 and P < 0.001, < 0.001, 0.001, and 0.035, respectively), and the monocyte/PMN ratio was correlated with CRP. Unlike in CD patients, no correlation was observed between the period from biologic discontinuation and relapse.

**Table 3:** Correlation analysis between monocyte and variables at the time of biologic discontinuation using Pearson correlation analysis.

Crohn’s disease (N = 159)		Variables at biologic discontinuation								
Monocyte		Duration of biologics treatment	Hematocrit	Albumin	ESR	CRP	Calprotectin	PCDAI	SES-CD	Time to relapse after D/C
**Percentage of WBC**	Pearson coefficient	-0.022	-0.145	-0.326^**^	0.299^**^	0.314^**^	0.371^**^	0.088	0.318^**^	-0.283^**^
	P-value	0.781	0.069	**<0.001**	**<0.001**	**<0.001**	**0.005**	0.272	**<0.001**	**0.001**
**Absolute count**	Pearson coefficient	0.003	-0.119	-0.344^**^	0.339^**^	0.379^**^	0.385^**^	0.168^*^	0.422^**^	-0.107
	P-value	0.973	0.134	**<0.001**	**<0.001**	**<0.001**	**0.003**	**0.034**	**<0.001**	0.236
**Monocyte/PMN ratio**	Pearson coefficient	-0.125	-0.230^**^	-0.346^**^	0.292^**^	0.288^**^	0.353^**^	0.197^*^	0.242^**^	-0.031
	P-value	0.115	**0.004**	**<0.001**	**<0.001**	**<0.001**	**0.007**	**0.013**	**0.002**	0.729
**Ulcerative colitis (N = 29)**		**Variables at biologic discontinuation**								
**Monocyte**		**Duration of biologics treatment**	**Hematocrit**	**Albumin**	**ESR**	**CRP**	**Calprotectin**	**PUCAI**	**MES**	**Time to relapse after D/C**
**Percentage of WBC**	Pearson coefficient	0.344	0.024	0.007	0.193	0.256	-0.070	-0.064	-0.201	-0.300
	P-value	0.067	0.903	0.970	0.317	0.180	0.762	0.743	0.295	0.114
**Absolute count**	Pearson coefficient	-0.076	0.092	-0.202	0.629^**^	0.721^**^	0.210	0.584^**^	0.392^*^	0.219
	P-value	0.694	0.634	0.293	**<0.001**	**<0.001**	0.361	**0.001**	**0.035**	0.254
**Monocyte/PMN ratio**	Pearson coefficient	0.316	0.353	-0.111	0.329	0.648^**^	0.050	0.259	0.148	-0.066
	P-value	0.095	0.060	0.565	0.082	**<0.001**	0.829	0.175	0.444	0.734

*P < 0.05, **P < 0.01 level (two-tailed).

Bold values mean statistically significant values with p-value <0.05.

### Role of monocytes as predictors of relapse with diagnostic capability

Several cases of relapse after biologic discontinuation after achieving deep remission have been reported. To evaluate this phenomenon, logistic regression analysis was used to identify the factors related to relapse (**Table 4**). Because the number of UC patients was small, only CD patients were analyzed. For univariate analysis, demographic data, such as age at the time of diagnosis and sex, were included, and SES-CD was included to evaluate the severity of the time of diagnosis. Factors regarding whether immunomodulators were being used at the time of biologic discontinuation and the duration of biologics treatment were also included, in addition to the monocyte values and other laboratory results assessing the activity of CD. Multivariate analysis was performed by selecting a variable with P < 0.1 (age, sex, duration of biologics treatment, monocyte percentage, monocyte/PMN ratio, albumin, and PCDAI score) in univariable analysis. As a result of multivariate analysis, the younger the age at diagnosis (odds ratio of 0.807 and P = 0.047), the shorter the duration of biologics use (odds ratio of 0.600 and P = 0.018), the higher the monocyte percentage (odds ratio of 2.012 and P < 0.001) and monocyte/PMN ratio (odds ratio of 4.320E+14 and P = 0.002), and the lower the albumin (odds ratio of 0.674 and P = 0.002), while the higher the PCDAI (odds ratio of 1.839 and P < 0.001) at the time of biologic discontinuation, the higher the probability of relapse after discontinuing the biologics.

**Table 4:** Multivariate logistic regression analysis for evaluation of risk factors for relapse after discontinuation of biologics in pediatric patients with Crohn’s disease. Multivariate analysis was performed by selecting a variable with P < 0.1 in univariable analysis.

Parameter	Univariate analysis				Multivariate analysis			
	P-value	Odds ratio	95% CI		P-value	Odds ratio	95% CI	
			Lower	Upper			Lower	Upper
**Age at diagnosis**	**0.056**	0.712	0.503	1.009	**0.047**	0.807	0.653	0.997
**Gender**	**0.022**	0.056	0.005	0.664				
**SES-CD** ^a^ **at diagnosis**	**0.056**	0.712	0.503	1.009				
**Immunomodulator status at D/C**	0.452	2.298	0.262	20.113				
**Duration of biologics treatment**	**0.030**	0.510	0.278	0.936	**0.018**	0.600	0.392	0.918
**Monocyte percentage of WBC**	**0.004**	2.018	2.006	2.030	**<0.001**	2.012	2.005	2.019
**Absolute count of monocyte**	0.689	1.207	0.480	3.037				
**Monocyte/PMN ratio**	**0.040**	68.698E+11	1.524	4.963E+23	**0.002**	4.320E+14	287572.010	6.489E+23
**Hematocrit at D/C**	0.151	0.838	0.659	1.067				
**ESR at D/C**	0.952	0.997	0.914	1.088				
**Albumin at D/C**	**0.003**	0.515	0.359	0.807	**0.002**	0.674	0.483	0.983
**CRP at D/C**	0.738	0.461	0.005	42.985				
**Fecal calprotectin at D/C**	**0.001**	2.073	1.353	3.177	**<0.001**	1.839	1.323	2.556
**SES-CD at D/C**	0.297	1.383	0.751	2.546				

a The simple endoscopic score for Crohn’s disease (SES-CD) assesses the size of mucosal ulcers, the ulcerated surface, the endoscopic extension, and the presence of stenosis.

b Pediatric Crohn’s disease activity index (PCDAI) score can range from 0 to 100, with higher scores signifying more active disease. A score of <10 indicates consistent with inactive disease, 11–30 indicates mild disease, and >30 indicates moderate-to-severe disease.

Bold values mean statistically significant values with p-value <0.05.

Since high monocyte values were evaluated as risk factors for relapse after biologic discontinuation, even after achieving deep remission, the diagnostic capability and cutoff value for predicting relapse with the monocyte values at the time of discontinuation were evaluated (**Figures 3**, **4**). **Figure 3** shows the ROC curve including patients who relapsed over all follow-up periods. In the UC patients, although the number of patients was small, the monocyte percentage and absolute monocyte count showed diagnostic capability (AUROC 0.725 and 0.765 and P = 0.042 and 0.017, respectively) with cutoff values of 6.65% and 490.50 count, respectively. Both the ROC curve including both CD and UC patients and the ROC curve including only CD patients had a significant P-value (all P < 0.001) and a high diagnostic capability (AUROC from 0.788 to 0.821). The cutoff value of the monocyte percentage was 7.05% and that of the absolute monocyte count was 449.93 count. Calprotectin, which is well known as a sensitive indicator for predicting relapse and CRP indicating the activity of inflammation, was also evaluated for their diagnostic values as a prediction of relapse using the ROC curve (**Supplementary figure 1**). In the case of CRP, it did not show a significant result as a diagnostic value because it had values in the normal range at the same time point when monocytes were collected. However, in the case of calprotectin showed diagnostic capability similar to that of monocyte results (AUROC = 0.760 with p value of 0.001).

Since the monocyte count at the time of discontinuation was found to be a reasonable predictor of early relapse than of distant relapse, **Figure 4** shows the ROC curve dividing patients who relapsed within 6 months and within 12 months after the discontinuation of biologics. If all three types of monocyte data were found to be meaningful, only the monocyte percentage was evaluated, since the data that can be viewed relatively quickly and as the data used in clinical practice is the monocyte percentage. When evaluating including relapses that occurred several times during the entire follow-up period, the faster the relapse occurred, the higher the monocyte percentage was. As the results, the cutoff value of the monocyte percentage, evaluated in relapsed patients within 12 months, was 7.65%. In the case of this period being less than 6 months, the cutoff value was 8.15%.

### Evaluation of rapid relapse and cumulative relapse probability using monocytes

**Figure 5:**
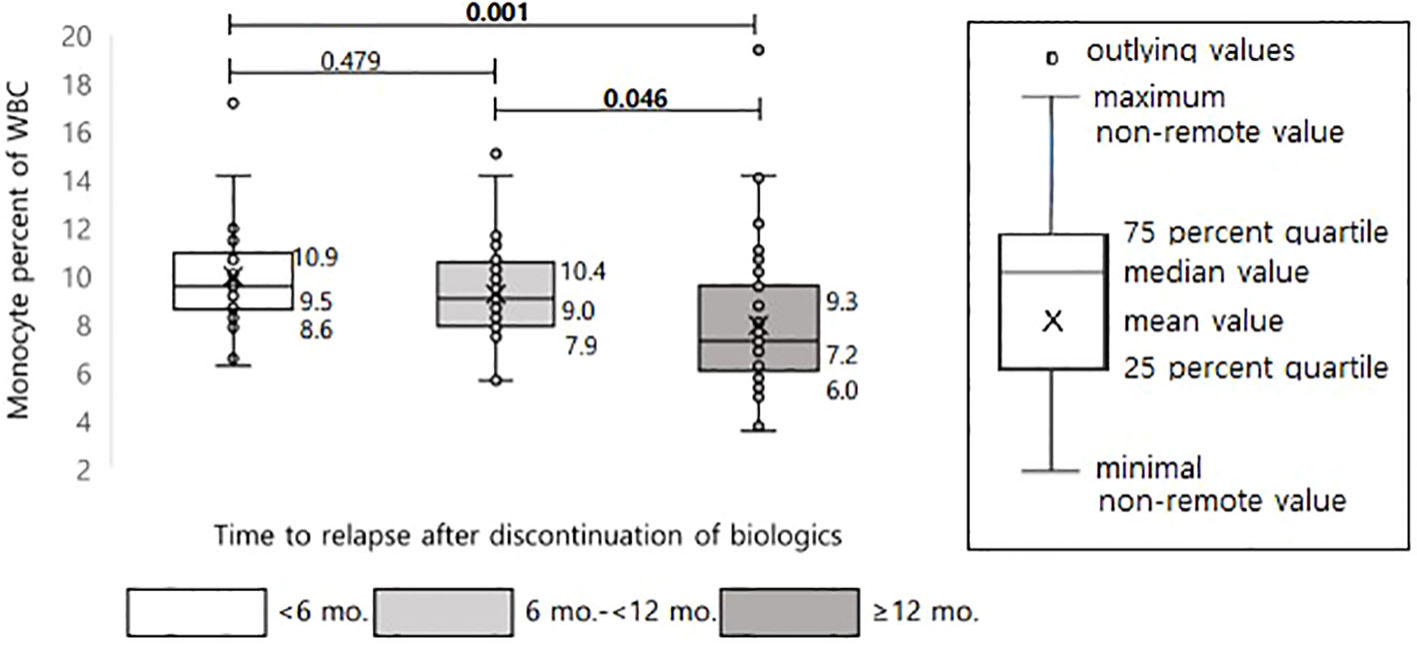
Differences in monocyte percentage of WBC according to time interval to relapse after discontinuation of biologics in pediatric patients with Crohn’s disease.

From the above results, it can be inferred that the higher the monocyte count at the time of biologic discontinuation, the faster relapse will occur. **Figure 5** shows the comparison of the median values of the monocyte percentage by dividing the relapsed groups within 6 months, at 6 months to 1 year, and after 12 months; the faster relapse occurred, the higher was the median value (median value of 9.5, 9.0, 7.2, respectively). The median values in the relapsed group within 6 months and the relapsed group after 12 months showed a significant difference with P = 0.001.

**Figure 6:**
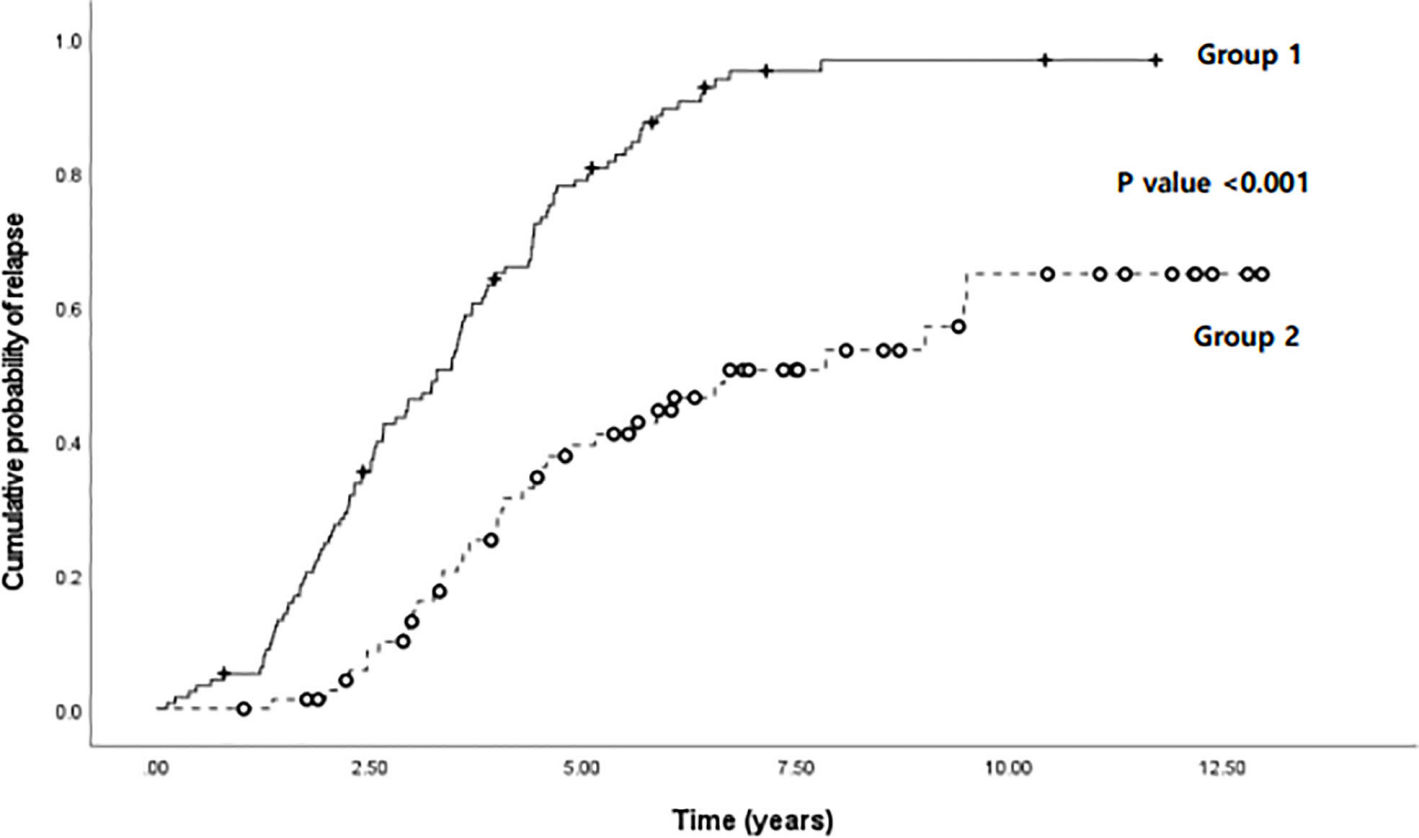
Comparison of cumulative probability of relapse according to time (years) between two groups: group 1, wherein the monocyte percentage was above the cutoff value (8.15%), and group 2, wherein the monocyte percentage was below the cutoff value, in pediatric patients with Crohn’s disease and ulcerative colitis.

In **Figure 6**, the results of the evaluation and comparison of the cumulative probability of relapse in the two groups are shown, including group 1, wherein the monocyte percentage at the time of biologic discontinuation was above the cutoff value (**Figure 3**), and group 2, wherein the monocyte percentage was below the cutoff value. Group 1 had a significantly higher cumulative probability of relapse than group 2 (P < 0.001).

## Discussion

This study retrospectively evaluated how monocytes could be interpreted clinically in patients with IBD using data collected from reviewing patients over a period of 19 years. Since many patients relapse after discontinuing biologics, even after achieving deep remission, and patients with monocytosis are often among those who achieve remission, we hypothesized that patients with monocytosis experience an inflammatory burden that results in relapse after the discontinuation of biologics. Therefore, the purpose of this study was to compare and evaluate monocyte counts in patients who experienced relapse after the discontinuation of biologics and those who did not.

Among our findings, monocytes at the time of discontinuation were found to be higher in the group of relapsed patients compared to the group in which relapse did not occur. These results indirectly show that if the monocytes are high at the time of biologic discontinuation, the probability of relapse increases. The severity of disease at the time of diagnosis was also evaluated under the assumption that relapse may be frequent due to disease severity at the time of diagnosis. However, only wide extent of the disease was significantly associated with more relapses in both CD and UC patients. Although the pathophysiology of IBD has not yet been fully elucidated, it is thought that widespread disease invasion may be more likely to experience relapse as it is associated with a broad immune response and a large decline in beneficial microbial species. In this regard, the wide extent of the disease is considered to be related to high monocyte counts, as the gut immune response is expected to occur within a wider range. In addition, the monocyte count at two months before relapse was noted to be elevated (**Supplementary Figure 2**), which suggests that the inflammatory burden becomes more severe in the upstream inflammation stage, subsequently leading to an actual inflammatory reaction.

A second important finding was the fact that monocytes were correlated with other laboratory results (albumin, ESR and CRP), fecal calprotectin, and endoscopic results (SES-CD or MES), indicating the biological activity underlying IBD. Although ESR and CRP were found in the normal range, fecal calprotectin was less than 200, and the SES-CD score was within the range 0–2. Furthermore, even within the normal range, the correlation with the monocyte count showed a slight upward trend when the inflammatory burden was present.

Monocytes were evaluated as a risk factor using logistic regression analysis to determine which factors act as risk factors for relapse at the time of biologic discontinuation. Other factors evaluated as risk factors (young age and fecal calprotectin) are already known to be risk factors for relapse (15–17). In particular, fecal calprotectin has been shown to be sensitive and useful as a tool to predict relapse in many studies (18–20). The authors also apply the fecal calprotectin a lot in clinical practice and when deciding the treatment direction. However, there are practical difficulties in frequently testing and evaluating fecal calprotectin in pediatric patients. In addition, it is difficult to obtain fecal calprotectin result on the same day as the test. Since it takes about 3-5 days to obtain the results of calprotectin, the clinician and the patients don’t know the results of calprotectin submitted on the day in outpatient clinic. In the case of blood tests, as these are performed during every visit to the hospital and the results are immediately available, it would be a useful test for predicting relapse like calprotectin. Thus, we evaluated the use of monocytes as a factor for the early detection of inflammatory burden using blood tests.

In patients administered biologics for a long period of time, unless they experience primary or secondary failure, remission has been found to be maintained for a long time, with relapse occurring less frequently (21). Thus, in the present study, a short duration of biologics treatment was evaluated as a risk factor for relapse after the discontinuation of biologics. Although clinical opinion on when to discontinue biologics remains a controversial topic, a recent review article argues for the need for individualized biologic discontinuation after sufficient observation, and recommends discontinuation in patients with a sufficiently long clinical, biological, and endoscopic remission period (22, 23). Although WBC has been evaluated as a risk factor for relapse in previous studies, these results are encouraging as this is the first study to evaluate monocytes at the time of discontinuation of biologics as a risk factor for relapse.

Previous studies have found that monocytes are a factor with sufficient potential as a predictor of relapse. Thus, in the present study, attempts were made to determine the cutoff value of monocytes that predicts relapse. Although monocytes were evaluated in three ways (the percentage of monocytes in the WBC count, the absolute monocyte count, and the monocytes/PMN ratio), and all three values were significant, we believe that the monocyte percentage in the WBC count is the easiest factor to use in actual clinical practice. In addition, considering that it is unrealistic that the results of blood tests at a specific period predict relapse in the distant future, the diagnostic capability of the monocyte percentage in patients who experienced rapid relapse within 6 months and within 12 months were evaluated separately (**Figure 4**). As a result, the monocyte percentage cutoff value was found to be higher when relapse within 12 months was included, compared to when relapse of the entire period was included. Furthermore, the cutoff value was found to increase in relapsed patients within 6 months compared to those who relapsed within 12 months. As shown in **Figure 5**, the actual monocyte values were found to be higher in patients who experienced rapid relapse. The monocyte percentage cutoff value for predicting relapse within 6 months was 8.15%, which was outside the normal range. Therefore, these results indicate that a monocyte percentage over 8.15% after biologic discontinuation is a predictor for rapid relapse within 6 months. Although there are many theoretical studies on the mechanisms underlying the role of monocytes in chronic inflammatory diseases (6, 7, 24, 25), the present study is the first to attempt to identify cutoff values using clinical data. Since fecal calprotectin is also being actively used in clinical practice as a sensitive marker for predicting relapse, we evaluated the ROC curve with fecal calprotectin. The fecal calprotectin test has been conducted in Korea since 2017, and the sample size is smaller than that of monocytes, so it is difficult to make a complete comparison with the monocyte results. Nevertheless, when evaluating the sensitivity and specificity of monocyte and fecal calprotectin, the sensitivity of monocytes was high at 84.4%, but the specificity was low. On the other hand, calprotectin had high specificity (85.7%) but low sensitivity. Based on our results, if monocytes are high, there is a high possibility of relapse, so it is necessary to carefully monitor the patient. For calprotectin, as in our results, not all high outcomes are predictive of relapse. This is because the level of calprotectin is high even in the case of gastrointestinal or anal bleeding, and the type of food eaten is also greatly affected. Therefore, when calprotectin is high, it is necessary to evaluate other causes together with bloody stool and no anal lesions. Calprotectin within the normal range can be used as a tool to exclude relapse.

Based on the cutoff value of 8.15%, 188 patients in the total patient group were evaluated by dividing the dataset into two groups, one with patients above the cutoff value of the monocyte percentage and one with patients below it, at the time of biologic discontinuation. As a result, patients who discontinued biologics at a monocyte value higher than the cutoff value showed a significantly higher cumulative relapse (**Figure 6**). In addition to supporting the above results, this finding also shows that the probability of relapse increases if biologics treatment is discontinued when the monocyte value is high. As shown in the graph, relapse also occurred in patients with monocyte values below the cutoff value. Thus, a high monocyte value alone does not explain all relapses. However, we can still argue that the discontinuation of biologics is not recommended, at least in patients with high monocyte values.

Collectively, our findings suggest that it is necessary to evaluate monocyte counts, along with the patient’s symptoms, ESR, CRP, fecal calprotectin, and endoscopy results, when deciding whether to discontinue the biologics. Theoretically, as biologics inhibit cytokines and inflammatory markers (ESR and CRP) are in normal range as the result of downstream inflammatory process, there is a need to evaluate whether there is an inflammatory burden at higher levels. In the present study, patients with high monocyte counts experienced a faster relapse within 6 months, wherein the monocyte percentage cutoff value predicting relapse within 6 months was 8.15% of the WBC count. These results suggest that biologics should not be discontinued in patients with a monocyte percentage above 8.15%, even if deep remission is achieved.

The main limitation of this study is its retrospective design in a single tertiary center. Therefore, there may be selection bias, in addition to confounding variables that were not considered. To minimize these biases and maintain the objectivity of the data, the three authors used the same criteria to collect data separately. In addition, as this is not a prospective design, some data do not precisely match the timing of the blood tests for infrequently performed tests, such as endoscopy and fecal calprotectin. However, despite these inherent limitations, this study is the first (even while considering all adult studies) that proposes the clinical application of monocytes for the early prediction of disease, based on immune mechanisms and clinical data from patients with IBD. In addition, the number of patients was relatively large for a study using pediatric patients, and both CD and UC patients were included. We believe that this study plays an important role as a preliminary study that provides additional criteria in the decision to discontinue biologics in the future. As a future research direction, we are planning a prospective study on monocytes in IBD patients, as well as a study comparing the relapse predictive properties of monocytes and fecal calprotectin.

## Conclusions

In this study, patients with a high monocyte percentage were found to have a faster rate of relapse within 6 months. Theoretically, since biologics inhibit cytokines, inflammatory markers (ESR and CRP) are in the normal range as a result of downstream inflammatory processes, it will be necessary to evaluate whether there is an inflammatory burden at higher levels. The results presented in this study indicate that it will be necessary to check monocyte abundance, along with patient symptoms, ESR, CRP, fecal calprotectin, and endoscopy results, when deciding whether to discontinue biologics in patients with IBD. Herein, a monocyte percentage over 8.15% in blood tests at the time of discontinuation was noted to be associated with a high probability of relapse within 6 months, even when deep remission was achieved. Therefore, it is not recommended that patients discontinue biologics if they have a monocyte percentage > 8.15%, even if deep remission is confirmed.

## Data availability statement

The raw data supporting the conclusions of this article will be made available by the authors, without undue reservation.

## Ethics statement

The studies involving human participants were reviewed and approved by Clinical Research Ethics Committee of Samsung Medical Center (IRB file no. SMC 2022-04-062). Written informed consent to participate in this study was provided by the participants’ legal guardian/next of kin.

## Author contributions

Conception or design: MJK. and YHC Acquisition, analysis, or interpretation of data: YK and YZK. Drafting the work or revising: YK and MJK. Final approval of the manuscript: YK and MJK. All authors contributed to the article and approved the submitted version.

## Funding

This study was supported by the National Research Foundation of Korea (NRF) grant, funded by the Ministry of Science and ICT (MSIT) of Korea (no. 2022R1F1A1073691).

## Conflict of interest

The authors declare that the research was conducted in the absence of any commercial or financial relationships that could be construed as a potential conflict of interest.

## Publisher’s note

All claims expressed in this article are solely those of the authors and do not necessarily represent those of their affiliated organizations, or those of the publisher, the editors and the reviewers. Any product that may be evaluated in this article, or claim that may be made by its manufacturer, is not guaranteed or endorsed by the publisher.

## Abbreviations

CD, Crohn’s disease; UC, ulcerative colitis; IBD, inflammatory bowel disease; IFX, infliximab; CR, clinical remission; PCDAI, pediatric Crohn’s disease activity index; PUCAI, pediatric ulcerative colitis activity index; ESR, erythrocyte sedimentation rate; CRP, C-reactive protein; ER, endoscopic remission; SES-CD, simple endoscopic score for Crohn’s disease; MES, Mayo endoscopic subscore; ROC, receiver operating characteristic; 5-ASA: 5-aminosalicylic acid.
