# Supplementary material for: Increased monocyte abundance as a marker for relapse after discontinuation of biologics in inflammatory bowel disease with deep remission
Source: Front Immunol. 2022 Nov 1;13:996875. doi: 10.3389/fimmu.2022.996875 (PMC9664214; doi:10.3389/fimmu.2022.996875)
Supplement: Supplementary file 1 [file DataSheet_1.pdf]

## Supplementary Material

### Supplementary Figures

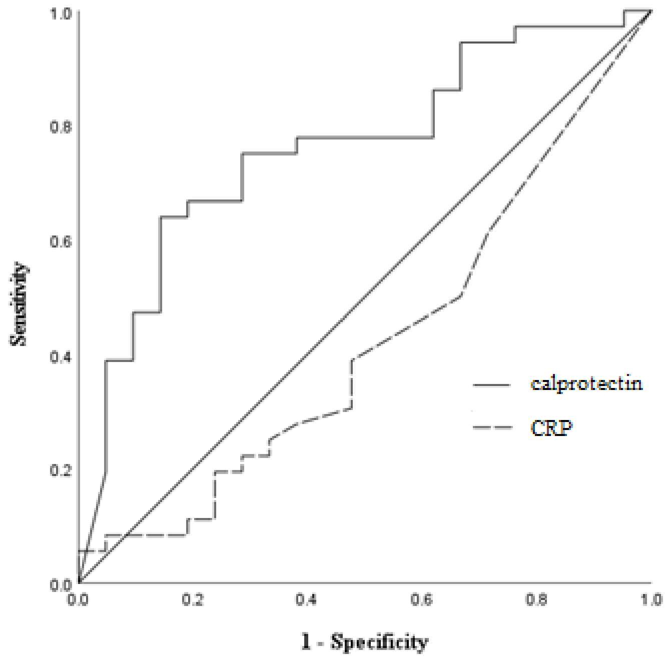


| | AUROC | P-value | 95% C.I. | | Cutoff value | Sensitivity | Specificity | (+) likelihood ratio | (-) likelihood ratio |
|---|---|---|---|---|---|---|---|---|---|
| | | | Lower | Upper | | | | | |
| **Calprotectin** | 0.760 | 0.001 | 0.631 | 0.889 | 67.7 | 63.9% | 85.7% | 4.46 | 0.42 |
| **CRP** | 0.421 | 0.325 | 0.266 | 0.576 | 0.44 | 35.5% | 80.0% | 1.78 | 0.81 |

**Supplementary Figure 1.** Diagnostic capabilities with area under operating characteristic curve of the fecal calprotectin and C-reactive protein for assessing relapse.

(a)

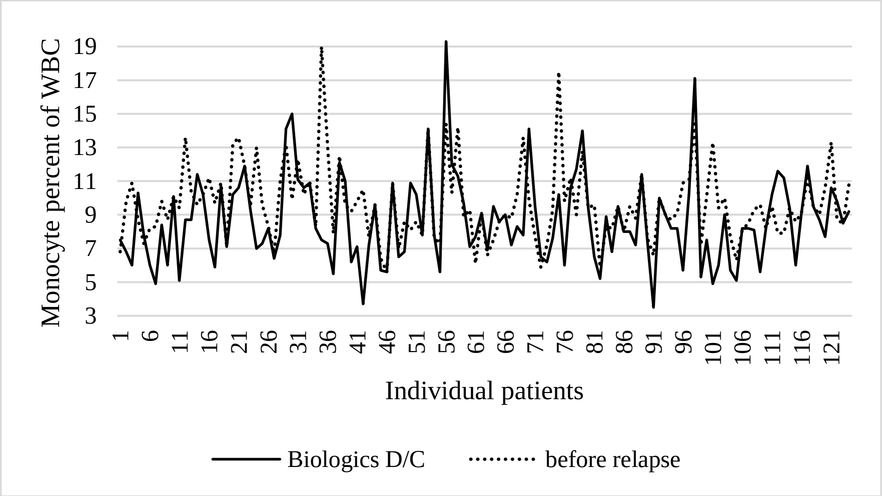


(b)

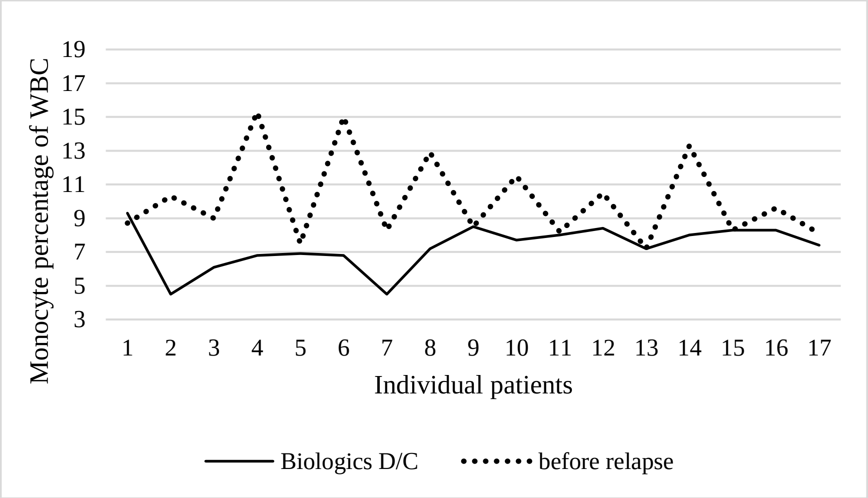


| | At the time of biologics D/C | | Two months prior to relapse | | P-value | |
|---|---|---|---|---|---|---|
| | CD | UC | CD | UC | CD | UC |
| Monocyte percentage of WBC | 8.65 ± 2.60 | 7.29 ± 1.31 | 9.67 ± 2.28 | 10.13 ± 2.58 | **0.001** | **<0.001** |

**Supplementary Figure 2.** Change in the percentage of leukocyte monocytes in an individual patient from the time of biologic discontinuation to two months prior to relapse in patients with (a) Crohn’s disease and (b) ulcerative colitis.

D/C; Discontinuation
